# Water Soluble‐Nitrogenous Secondary Metabolites From *Talaromyces annesophieae* MD2 Exhibit Anti‐Bacterial, Anti‐Cancer, Anti‐Oxidant, and Cytoprotective Activities

**DOI:** 10.1002/mbo3.70367

**Published:** 2026-07-19

**Authors:** Meryem Doymus‐Yilmaz, Huseyin Aksit, Mesut Taskin

**Affiliations:** ^1^ Vocational School of Health Services of Hinis Ataturk University Erzurum Türkiye; ^2^ Department of Analytical Chemistry, Faculty of Pharmacy Erzincan Binali Yildirim University Erzincan Türkiye; ^3^ Department of Molecular Biology and Genetics, Faculty of Science Ataturk University Erzurum Türkiye; ^4^ Institute of Medicine, Vaccine and Biotechnology Ataturk University Erzurum Türkiye

**Keywords:** antibacterial activity, anticancer activity, bioactive compounds, fungal biotechnology

## Abstract

This study focused on biological activities and chemical characterization of secondary metabolites from the fungus *Talaromyces annesophieae* MD2 (GenBank: PQ252671). The dichloromethane extract (DCM‐E) of its culture supernatant was fractionated using chromatographic techniques. Initially, three main fractions were first prepared from DCM‐E. Subsequently, the selected active fraction 2 was subfractionated into six subfractions. The subfraction 3 demonstrated high water solubility and strong antibacterial activity against both gram‐positive and gram‐negative bacteria (MIC values of 105.41, 82.76, 71.92, and 70.38 µg/mL against *B. cereus, S. aureus, E. coli*, and *K. pneumoniae*, respectively). It exhibited selective anticancer activity through regulation of autophagy‐ and apoptosis‐related pathways without affecting healthy cells (IC_50_ values of 7.65, 3.76, 10.76 µg/mL against HT‐29, DU‐145 and SH‐SY5Y, respectively). Furthermore, it exhibited DPPH and ABTS radical‐scavenging activities (48.3% and 44.1% at the concentration of 100 μg/mL, respectively) and protected dermal fibroblasts against H_2_O_2_–induced toxicity. Structural analyses elucidated that the subfraction 3 contained four nitrogenous polar compounds. Three compounds (C_5_H_3_O_4_N_8_, C_15_H_25_O_4_N_4,_ and C_23_H_21_N_4_) were suggested to be alkaloid‐like or nitrogen‐rich heterocyclic secondary metabolites, while the remaining compound (C_9_H_9_O_4_N) was probably an aminobenzoic acid‐type or heteroaromatic carboxylic acid derivative. This is the first report on bioactive metabolites of *T. annesophieae*. Due to multifunctional biological activities, its metabolites can find application in dermocosmetics and pharmacology.

## Graphical abstract

The dichloromethane extract (DCM‐E) was prepared from the culture supernatant of the fungus *Talaromyces annesophieae* MD2 was fractionated using chromatographic techniques. The subfraction 3 was determined to contain four major nitrogenous compounds and exhibit antibacterial, anticancer, antioxidant, and cytoprotective activities.

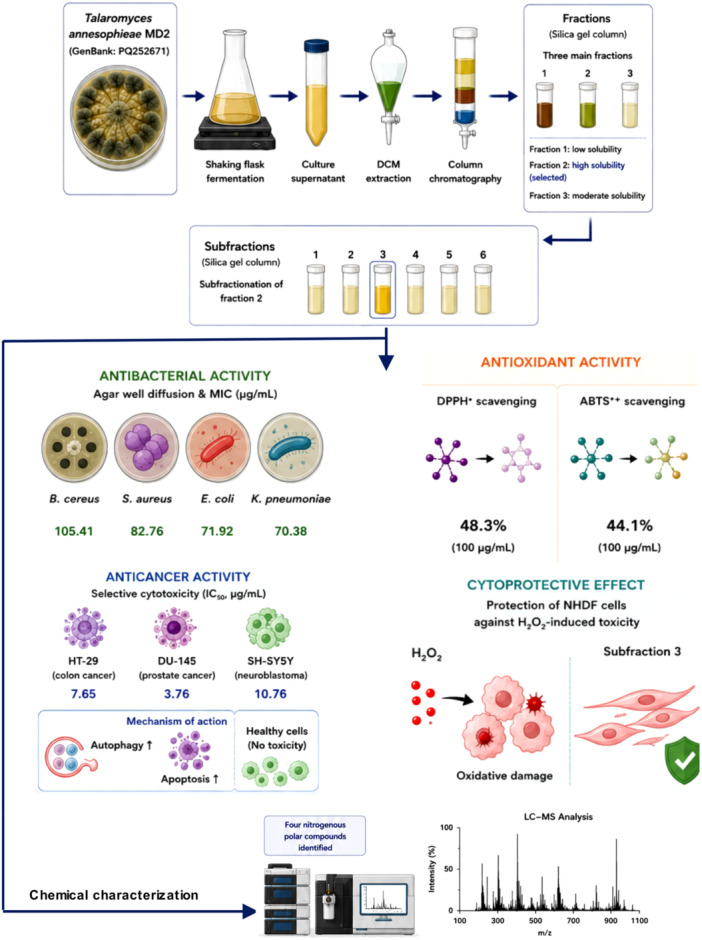

## 1 Introduction

Microorganisms, namely bacteria, fungi and microalgae, are one of the most known natural sources of bioactive metabolites (Arslan et al. 2024; Esim et al. 2024). In the literature, it has been documented that 553 new fungal bioactive metabolites were discovered in 2003, and 907 in 2024 (Shi et al. 2024; Bao et al. 2025). However, it is estimated that the total number of fungal bioactive metabolites discovered to date is around 15000‐20000, with the majority being secondary metabolites (Conrado et al. 2022).

The secondary metabolites sourced from fungi can be structurally classified as alkaloids, benzopyranones, quinones, peptides, phenols, quinones, isocoumarins, flavonoids, steroids, terpenoids, tetralones, xanthones, and nitrogen‐rich heterocyclic metabolites (Anh et al. 2022; Conrado et al. 2022; Hashem et al. 2023; Basappa et al. 2023; Shi et al. 2024). These metabolites exhibit diverse bioactive properties, such as anti‐cancer, antioxidant, anti‐inflammatory, antimicrobial, antidiabetic, and immunomodulatory properties, which make them interesting for pharmaceutical, nutraceutical and cosmeceutical industries (Taufiq and Darah 2018; Ujam et al. 2019; Gutiérrez and González 2019; Conrado et al. 2022; Hashem et al. 2023; Zhang et al. 2022; Arslan et al. 2025a).

In the literature, it has been documented that filamentous fungi such as *Aspergillus, Penicillium* and *Talaromyces* are the most interesting producers of bioactive metabolites (Bladt et al. 2013). *Talaromyces* is one of the large fungal genera in the phylum *Ascomycota*, family *Trichocomaceae*, consisting of 171 species. It is characterized by the production of creamy, yellow, white, pinkish or reddish ascomata and yellow ascospores. The genus *Talaromyces* is an important fungal genus that can be isolated from soil, plants, sponges and foods. Species in the *Talaromyces* group in particular have the potential to be used in pharmaceutical, medical, industrial and biotechnological fields (Lan and Wu 2020).

The members of the genus *Talaromyces* can synthesize a variety of bioactive metabolites such as polysaccharides, pigments, phycobilins, mycosporins, phenolics, alkaloids, peptides, terpenes, terpenoids, flavonoids, lactones, phenolics, steroids, and tetralones (Zhai et al. 2016; Gutiérrez and González 2019; Rustamova et al. 2020; Yazici et al. 2020; Singh et al. 2021). For instance, some studies have revealed that *Talaromyces* species such as *T. flavus, T. pinophilus, T. trachyspermus, T. stipitatus, T. amestolkiae* are able to produce secondary metabolites exhibiting health beneficial activities such as antioxidant, anticancer, α‐glucosidase inhibitory, antiviral and antibacterial (Frisvad et al. 2013; Kumla et al. 2014; Matsunaga et al. 2015; Zhai et al. 2015; Zhai et al. 2016; Chen et al. 2016; Cai et al. 2017). On the contrary, to the best of our knowledge, there is no study on the bioactive metabolites of locally isolated *T. annesophieae* MD2. Considering this gap in the literature, the present study focused on the biological activities and chemical characterization of secondary metabolites of *T. annesophieae* MD2.

## 2 Materials and Methods

### 2.1 Isolation of Filamentous Fungi

Pine forest soil samples was collected from different localizations of Erzurum (Turkey), namely Pasinler (40°05'23.9“N41°44'07.3“E), Tortum (40°28'38.0“N 41°16'21.4“E), Yakutiye (39°54'27.3“N 41°14'33.4“E) and Palandöken (39°52'35.5“N 41°18'48.0“E). The samples were transported to laboratory for further processing. Soil samples were diluted with sterile‐saline water and 1 mL of diluted sample spread on petri dishes containing potato dextrose agar (PDA). Petri dishes were incubated in an incubator at 30°C for 120 h (Taskin et al. 2016; Kayar et al. 2022). After an incubation period the colonies growing on petri dishes were examined under a light microscope, and the colonies thought to be filamentous fungi were purified by subculture on PDA.

### 2.2 Antimicrobial Activities of Culture Filtrates of Fungal Isolates

In this stage of the study, the antimicrobial activity of the culture supernatants of the isolates was tested. However, the cultures were not subjected to extraction with any organic solvent, and the culture supernatants were used directly in the antimicrobial activity experiments. The isolates were first activated on PDA medium at 30°Cfor 6 days. At the end of the growth period, one disc (1 × 1 cm) was excised from the fungus mycelia on PDA with a sterile scalpel and was then inoculated into 100 mL of sterile potato dextrose broth (PDB) in 250 mL erlenmeyer flasks. Following inoculation, the flasks were placed in an orbital shaker and were then left to the incubation at 130 rpm and 30°C for 10 days (Zhang et al. 2022). At the end of incubation, the cultures of fungal isolates were first filtered with filter paper and the obtained supernatant was centrifuged at 6000 rpm for 10 min.

Antimicrobial activity of the supernatants was tested according to agar well diffusion method (Wiegand et al. 2008) against *Escherichia coli* O157:H7 ATCC 43894 (Gram‐negative bacterium) and *Staphylococcus aureus* ATCC 25923 (gram‐positive bacterium). To do this, test bacteria were first cultured in Nutrient Broth (NB) medium for 24 h, and at the end of this period, the concentration of each culture was adjusted to 0.5 McFarland. Following these procedures, 0.1 mL of each sample was spread on Nutrient Agar (NA) with a sterile cotton swab. Subesequently, 40 µL of the culture filtrate were added to wells, which were opened on NA with the help of a piercer. After the petri dishes were incubated at 37°Cfor 24 h, diameter of clear zone around wells was measured. The isolate causing the largest zone on well was selected and used for the subsequent stages of the study.

### 2.3 Molecular Identification of the Most Potent Fungal Isolate

The molecular identification of the selected fungal isolate was performed based on ITS (Internal Transcribed Spacer) rRNA gene sequencing analysis. For this purpose, the fungal isolate was initially grown in 250 mL flask containing 100 mL of PDB on shaking incubator (at 25°C for 72 h). The total genomic DNA of the isolate was isolated from cell biomass using Promega wizardR genomic DNA purification kit. The primer used were ITS1 5′TCCGTAGGTGAACCTGCGG3′ forward and ITS4 5′TCCTCCGCTTATTGATATGC3′ reverse (Kumar and Shukla 2005). The ITS sequence of the isolate was compared with the fungal sequences retrieved from GenBank using the BLAST‐n analysis. The resulting sequences, which were downloaded from the company's website, were processed and analyzed using BioEdit software. The obtained sequences were then compared with other fungal sequences available in GenBank (http://blast.ncbi.nlm.nih). A phylogenetic tree was subsequently constructed using MEGA4 software, based on the ITS sequences of the strain most closely related to the isolates.

### 2.4 Antimicrobial Activity of DCM and Etoac Extracts of Culture Supernatant

The selected isolate was inoculated in 250 mL erlenmeyers containing 100 mL PDB and then allowed to grow at 30°C and 130 rpm for 10 days. Following the incubation, filtration and centrifugation processes were applied to the culture and the obtained supernatant was subjected to extraction process with different organic solvents (DCM and EtOAc, 1:1 ratio). After extraction, the organic phase was separated and the solvent was evaporated with an evaporator at 35°C–40°Cuntil the final volume was 1 mL. DCM and EtOAc were also used separately as negative solvent controls. Then, the antimicrobial activity of the extract was tested. Based on the obtained results, the extract exhibiting higher antibacterial effectiveness against two test bacteria was determined and the subsequent stages of the study were carried out with this extract.

### 2.5 Fractionation of DCM Extract

The dried DCM partition of the fungal culture broth was initially subjected to size‐exclusion chromatography on a Sephadex LH‐20 column eluted with 100% methanol. A total of 27 fractions were collected. Based on their Thin‐Layer Chromatography (TLC) profiles on silica gel plates, fractions showing similar chemical behaviors were pooled to afford three major sub‐fractions (Fr. A, Fr. B, and Fr. C).

These three major fractions were evaluated for their initial antimicrobial activities against *Escherichia coli* and *Staphylococcus aureus* using the agar well diffusion method. The screening was performed at concentrations of 100 µg/mL to monitor the activity distribution and determine the final antimicrobial profiles. The most active sub‐fraction (Fr. B, 5.0 g) was selected for purther separation process and dissolved in deionized water. A minor insoluble portion was filtered out, and the clear filtrate was directly loaded onto a pre‐packed C18 reversed‐phase flash column (40 g). Elution was conducted using a step gradient of deionized water and methanol (150 mL per step) with the following ratios (v/v): 10:0, 9:1, 8:2, 7:3, 6:4, and 0:10, yielding six distinct sub‐fractions (Sub‐fr. 1–6). The methanol in the collected sub‐fractions was removed under reduced pressure at 40°C, and the remaining aqueous phases were lyophilized. The final dried powders were stored for subsequent antimicrobial assays and chemical characterization. Subsequently, these six sub‐fractions were dissolved in sterile distilled water and evaluated for their antibacterial activities at concentrations of 100 µg/mL against *E. coli* and *S. aureus*. Among the tested samples, Sub‐fr. 3 (eluted with 8:2 deionized water:methanol) exhibited the highest potency and was prioritized for final chemical characterization. In short, the selection of the most active fraction and subfraction in this study was based on the measurement of antibacterial activity.

### 2.6 Evaluation of Biological Activities of the Most Potent Subfraction

#### 2.6.1 Antimicrobial Activity of the Most Potent Subfraction

At this stage of the study, the antimicrobial effectiveness of the subfraction was evaluated against *E. coli* ATCC 43894 and *S. aureus* ATCC 25923, as well as *Bacillus cereus* ATCC 11778 and *Klebsiella pneumoniae* ATCC 13883.

In agar well diffusion method, five different concentrations of the selected subfraction (50–150 µg/mL in distilled water) were tested against these pathogens, as described above.

In the MIC test, the stock solution (5 mg/mL) of the selected subfraction was first prepared in distilled water. It was serially diluted in distille water to obtain final test concentrations (25, 50, 100, 200, and 400 μg/mL). Test bacteria were activated on NA and incubated at 37°C for 24 h. Colonies were suspended in sterile saline and the density of suspensions was adjusted to 0.5 McFarland standard (1 × 10^8^ CFU/mL). MIC determination was performed according to microdilution protocol in 96 well microtiter plate. For this purpose, 100 μL of the subsfraction sample (the sample at the concentration of 25, 50, 100, 200, or 400 μg/mL) and 100 μL of bacterial inoculum were mixed in each well. Control well contained inoculum (100 μL) and distille water (100 μL). After plates were aerobically incubated at 37°C for 24 h, bacterial growth was monitored at 600 nm using a microplate reader. The MIC was defined as the lowest extract concentration that causes complete inhibition of bacterial growth.

#### 2.6.2 Evaluation of Anticancer Activity of the Most Potent Subfraction

Cytotoxicity of subfraction was tested on three cancer cell lines **[**human colon cancer cell line (HT‐29), human neuroblastoma cancer cell line (SH‐SY5Y), human prostate cancer cell line (DU‐145)] and one healthy cell line [lung fibroblast cell line (MRC‐5)]. HT‐29, SH‐SY5Y, and DU‐145 cell lines were cultured in high‐glucose Dulbecco's Modified Eagle Medium (DMEM), supplemented with 10% fetal bovine serum (FBS), 1% Penicillin‐Streptomycin (100 U/mL penicillin, 0.1 mg/mL streptomycin), and 1% Amphotericin B. MRC‐5 cell line was cultivated in low‐glucose DMEM supplemented with the same concentrations of FBS, antibiotics, and antifungal agents under identical incubation conditions. All cells were cultured at 37°Cin a %5 CO_2_ atmosphere. Cells were subcultured upon reaching 70%–80% confluency.

Cells were seeded into 96‐well plates at a density of 10 × 10^3^ cells per well in 100 µL of complete medium.

Cells from all lines were treated with various concentrations (1.25–20 μg/mL) of the test fraction diluted in complete medium. After 24 h of treatment, 3 µL of WST‐1 reagent was added to each well and incubated for at least 3 h. Absorbance was measured at 450 nm using a microplate reader (SpectraMax, Molecular Devices).

The mechanisms underlying the anticancer effect of the most potent subfraction were investigated according to the changes in expression levels of apoptosis‐, autophagy‐, and inflammation‐related genes. For this purpose, following 24 h of exposure to the subfraction, cells were harvested and total RNA was extracted from each sample using a commercial RNA isolation kit (Hibrigen, Turkey) according to the manufacturer's instructions. The purity and concentration of the RNA samples were determined using spectrophotometric analysis, and only RNA with acceptable purity (A260/A280 ratio between 1.8 and 2.0) was used for downstream applications. Complementary DNA synthesis was performed using a commercial cDNA synthesis kit (Ampigene®, Enzo Life Sciences). Quantitative real‐time PCR (qRT‐PCR) was used to assess the expression levels of caspase‐3, caspase‐8, caspase‐9, caspase‐1, NF‐κB, beclin‐1, Bax, and Atg14. The gen expressions were conducted by using SYBR Green Master Mix (2X; Thermo Fisher Scientific) and β‐actin was used as an housekeeping gene. The amplified products were measured using amplification curve analysis. All data were analyzed using the 2^–∆∆CT^ method (Livak and Schmittgen 2001). The primer sequences used for gene expression analysis are listed in Supplementary Table 1.

#### 2.6.3 Evaluation of In Vitro Antioxidant Activity of the Most Potent Subfraction

The 1,1‐diphenyl‐2‐picrylhydrazyl (DPPH) radical scavenging activity of the selected subfraction was tested according to the method described in earlier studies (Blois 1958; Albayrak et al. 2026). For activity measurement, 0.9 mL of DPPH solution (0.05‐3.2 mg/mL) and 0.3 mL of subfraction solution (6.25–100 μg/mL) were mixed and this mixture was incubated for 30 min at room temperature in the dark. At the end of the incubation period, the absorbance value of the reaction mixture was measured at 517 nm. Deionized water (0.3 mL) was used as a control during the experiment, and vitamin C (ascorbic acid) solutions (6.25, 12.5, 25, 50 and 100 μg/mL) were used as positive controls. The DPPH radical scavenging potential was calculated using Equation 1.

The potential of subfraction 3 to scavenge 2,2′‐azino‐bis(3‐ethylbenzothiazoline‐6‐sulfonic acid) diammonium salt (ABTS) was evaluated according to the method suggested in the previous studies (Ji et al. 2021; Yildirim et al. 2026). In this method, 9.9 mg of potassium persulfate was mixed with 15 mL of 5.55 mmol/L ABTS aqueous solution and the solution was incubated at 25°C in the dark for 15 h to produce a blue‐green color. At the end of the incubation period, the solution was diluted with PBS (100 µmol/L, pH 7.4) until the absorbance value of the solution became 0.70 at 734 nm. The reaction mixture consisting of 1 mL subfraction (6.25–100 μg/mL) and ABTS solution (2 mL) was allowed for 25 min and after this period, the absorbance value of the mixture at 734 nm was measured. Ascorbic acid solutions prepared at the same concentrations were used as positive control. ABTS radical scavenging activity was calculated using Equation (1).

(1)

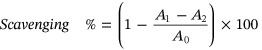




In the formula used, *A*_0_ represents the absorbance of the control solution (prepared with deionized water and DPPH/ABTS), *A*_1_ represents the absorbance of the solution prepared with DPPH/ABTS solution and subfraction/ascorbic acid solution. *A*_2_ represents the absorbance of the solution containing other components without DPPH/ABTS.

#### 2.6.4 In Vitro Cytoprotective Activity of the Most Potent Subfraction

Normal human dermal fibroblasts (NHDFs) were grown in PromoCell fibroblast growth medium enriched with l‐penicillin‐streptomycin and 10% fetal bovine serum (Gibco) at 37°C in a humidified environment containing 5% CO_2_. The growth medium was renewed every 2–3 days, and passaging was performed when the fibroblast cells reached 70%–80% density. To replicate the aging process, NHDFs were cultured overnight in a 96‐well plate using the growth medium at a density of 8000 cells per well. Subsequently, one group of NHDFs was treated with 300 μM hydrogen peroxide (H_2_O_2_), representing the toxicity group. The other groups of NHDFs was treated with different concentrations of the most potent subfraction (10–80 μg/mL) along with H_2_O_2_ (300 μM). In these group designed, the aim was to demonstrate the potential protective effect of the subfraction against H_2_O_2_ toxicity. In the control group, NHDFs were not treated with H_2_O_2_ and the subfraction. All groups were incubated in medium for 24 h (Quiles et al. 2022; Yildirim et al. 2026). After the incubation period, the medium was removed and the wells were washed with PBS. Cell viability was assessed using WST‐1.

### 2.7 Chemical Characterization of the Most Potent Subfraction

Chemical characterization of the subfraction determined to exhibit biological activity was performed using UV spectrophotometer, HPLC, GC‐MS, and LC‐MS/MS.

### 2.8 Statistical Analysis

Statistical analyses were performed using GraphPad Prism version 8.0.2 software (GraphPad Software, San Diego, USA). Data were expressed as mean ± standard deviation (SD). Statistical comparisons between groups were performed using one‐way analysis of variance (ANOVA) followed by appropriate post hoc tests for multiple comparisons. A p‐value of less than 0.05 was considered statistically significant. Statistical significance levels were defined as follows: *p* > 0.05 (ns; not significant), *p* < 0.05 *(* significant), *p* < *0.01 **(** highly significant), and p* < *0.001 (*extremely significant).

## 3 Results and Discussion

### 3.1 Isolation and Identification of Antibacterial Metabolites‐Producing Fungi

Fungi are known to produce various antibacterial metabolites such as polysaccharides, peptides, pigments, phenolic and alkoloids (Li et al. 2023; Hussein et al. 2022; Widayanti et al. 2019; Arslan et al. 2025a 2025b). The present study mainly focused on discovering a fungal strain capable of producing metabolites with antimicrobial activity. However, other biological activities of the target metabolite/metabolites were also investigated in this study. In this regard, a total of 40 fungal strains which exhibit different morphological properties and colorotion were isolated from four soil samples (Supporting Information S1: Figure S1). Their antibacterial effectiveness was first tested against two bacteria (Gram negative *E. coli*, and Gram positive *S. aureus)*. In the initial step, culture supernatants were directly used to test antibacterial activity. The agar well diffusion assay demonstrated that out of 40 isolates, the culture filtrates of three isolates (MD2, MD7 and MD11) possessed strong antimicrobial efficacy against *E. coli* and those of four isolates (MD2, MD8, MD12, MD24) did against *S. aureus* (Supporting Information S1: Figure S2, Table S2). As seen from these results, the culture filtrate of isolate MD2 exhibited antibacterial efficacy both *E. coli* and *S. aureus*. Based on these results, the isolate MD2 was chosen for further stages.

**Figure 1:**
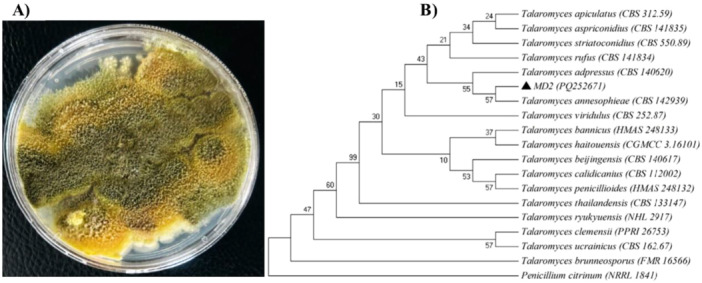
Colony morphology of the isolate MD2 on PDA (A) and its neighbor joining phylogenetic tree on the basis of 18S rRNA gene sequence (B). In phylogenetic tree, bootstrap values based on 1000 replications are listed as percentages at branching points. The accession numbers are given in parenthesis. Only bootstrap values > 50% are shown at nodes. The scale bar represented 1% divergence.

The isolate MD2 was found to develop mycelia with yellowish‐green to dull green tones on potato dextrose agar (Figure 1a). BLAST‐based evaluation of the 18S rRNA gene sequence of isolate MD2 against the GenBank database demonstrated a high degree of similarity to *Talaromyces* species. To resolve its phylogenetic position, a neighbor‐joining tree was constructed using 18S rRNA sequences from representative *Talaromyces* strains, with bootstrap analysis providing statistical validation. The analysis revealed that the MD2 (GenBank accession number: PQ252671) was closely related to *Talaromyces annesophieae* CBS 142939 (Figure 1b).

*T. annesophieae* is a relatively newly identified fungal species; therefore, there is limited literature available on this organisms (Visagie et al. 2024; Tsang et al. 2018; Chen et al. 2016). This species is generaly isolated from soil, plant or marine environments and has no pathogenity for humans (Tsang et al. 2018; Zhai et al. 2016). In the literature, it has been documented that some *Talaromyces* species such as *T. flavus*, *T*. *pinophilus*, *T. trachyspermus*, *T. stipitatus*, *T. amestolkiae* can synthesize different bioactive metabolites (Kumla et al. 2014; Frisvad et al. 2013; Matsunaga et al. 2015; Cai et al. 2017; Zhai et al. 2015; Chen et al. 2016); however, the bioactive properties of *T. annesophieae* metabolites have not been examined in the literature yet. Accordingly, we consider that exploring a bioactive metabolite of this species can contribute biotechnological and medicinal studies.

### 3.2 Antimicrobial Activity of Different Extracts of Culture Supernatant of *T. annesophiaeae* Md2

In this stage, the culture supernatant was extracted separately with dichloromethane (DCM) and nd Ethyl Acetate (EA), and the prepared extracts were then assessed for their antimicrobial activities against *E. coli* and *S. aureus*. The DCM and EA extracts‐induced zone diameters were determined to be 20 and 12 mm against *E. coli*, 17 and 10 mm against *S. aureus*, respectively. No inhibition was observed around well, in which DCM or EtOAc was used as negative solvent control (Supporting Information S1: Figure S3). These results indicated that DCM extract possessed higher antibacterial efficacy against both bacteria. Considering this finding, the following steps regarding with biological activities were conducted using DCM extract.

### 3.3 Fractionation of DCM Extract

**Figure 2:**
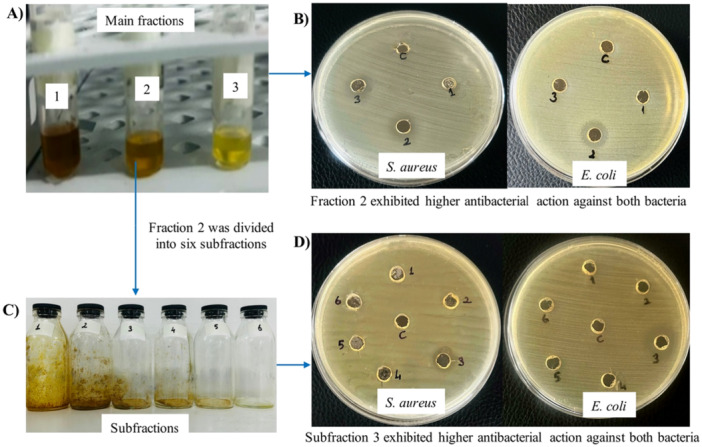
Fractionation of DCM extract by chromatographic methods. (A) Three main fractions prepared from DCM extract, B) the antibacterial effectiveness of three main fractions at a concentration of 100 µg/mL, C) six subfractions prepared from the active fraction (fraction 2), and D) antibacterial effectiveness of six subfractions at a concentration of 100 µg/mL. Antimicrobial activity of water soluble subfraction 3 (zone diameters of 13 and 20 mm against *S. aureus* and *E. coli*, respectively).

In this stage of the study, the fractions and subfractions were prepared from the DCM extract, and those with the highest antibacterial activity were selected for the next step. Experimental design of this stage is summarized in Figure 2.

As seen from Figure 2a, three main fractions (1–3) were first prepared from DCM extract. These fractions were then tested for their antibacterial effectiveness at a concentration of 100 µg/mL. Fraction 1 showed no antibacterial activity against either *E. coli* or *S. aureus*. Both fraction 2 and fraction 3 displayed an antibacterial action against *S. aureus*, while antimicrobial effect against *E. coli* was achieved with only fraction 2. These results indicate that only fraction 2 has a notable antibacterial potency against both bacteria (Figure 2b). Consequently, the fraction 2, which exhibited the strongest antibacterial activity, was selected for the subsequent purification stages. For this purpose, the fraction 2 was re‐separated into six different subfractions by chromatographic methods (Figure 2c).

Six new subfractions were then investigated for their antimicrobial activity against two bacteria at a concentration of 100 µg/mL; however, agar well difusion assay elucidated that only fraction 3 has a detectable antimicrobial action against both bacteria (Figure 2d). Based on this finding, the subsequent experiments were performed using the subfraction 3.

### 3.4 Biological Activities of Subfraction 3

#### 3.4.1 Antibacterial Activity of Subfraction 3

In the present study, the five different concentrations of the subfraction 3 (50, 75, 100, 125, and 150 µg/mL) were prepared using distilled water, and they were tested for antimicrobial effectiveness against two gram‐negative bacteria (*E. coli* and *K. pneumoniae*) and two gram‐positive bacteria (*S. aureus* and *B. cereus*). The antibacterial potential of this subfraction was found to increase as its concentration increased. For example, this fraction exerted the highest cytotoxicity effect on all test bacteria when used at the concentration of 150 µg/mL. At this concentration, inhibition zones were determined to be 21, 19, 23 and 22 mm against *S. aureus, B. cereus, E. coli* and *K. pneumoniae*, respectively (Supporting Information S1: Figure S4, Table 1). The MIC values of the fraction 3 were determined to be 105.41, 82.76, 71.92, and 70.38 µg/mL against *B. cereus, S. aureus, E. coli*, and *K. pneumoniae*, respectively (Table 1).

**Table 1:** Antimicrobial activity of subfraction 3.

Groups	Zone diameter (mm)			
	*S. aureus*	*B. cereus*	*E. coli*	*K. pneumoniae*
dH_2_O (Control)	NI	NI	NI	NI
50 µg/mL	NI	NI	NI	NI
75 µg/mL	NI	NI	NI	NI
100 µg/mL	14 ± 1.0	11 ± 1.0	15 ± 1.0	16 ± 1.0
125 µg/mL	18 ± 1.0	15 ± 1.0	19 ± 1.0	19 ± 1.0
150 µg/mL	21 ± 1.0	19 ± 1.0	23 ± 1.0	22 ± 1.0
MIC (µg/mL)	82.76	105.41	71.92	70.38

*Note:* Antimicrobial effectivenes of subfraction 3 was evaluated according to agar well diffusion and MIC assays.

#### 3.4.2 Anticancer Activity of Sub‐Fraction 3

To date, numerous studies have manifested that fungal metabolites or extracts with antimicrobial activity have also other bioactive properties, including anticancer, antioxidant, and anti‐aging activities (Al‐Saleem et al. 2022; Ansari et al. 2013). For example, Niazi et al. (2023) demonstrated that the EA extract from *A. niger* possessed anti‐bacterial, antifungal, and anticancer activities. In a separate study Abdel‐Hady et al. (2016), a purified metabolite from *P. islandicum* was shown to have anticancer and anti‐bacterial activities without anti‐fungal activity. Consequently, the other biological activities of the fraction 3 were also tested in this study.

**Figure 3:**
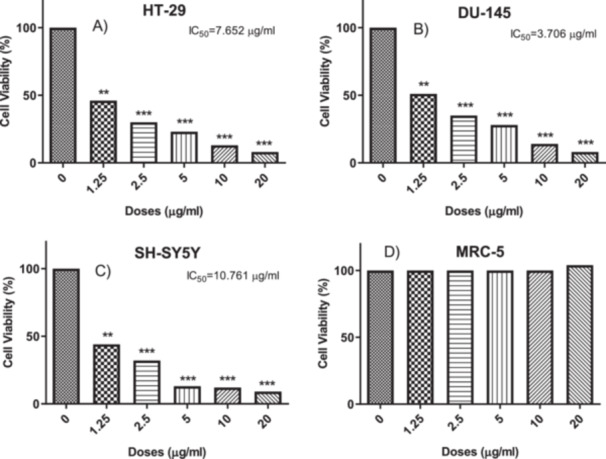
Anticancer potential of fraction 3 on different cancer cell lines. (A) HT‐29, (B) DU‐145, (C) SH‐SY5Y cancer cell lines and (D) MRC‐5 cell line. Two independent experiments were conducted and measurements were made in at least three replicates (*n* = 6) and presented as mean ± SD. Data are given as mean ± standard deviation and *n* = 3. **p < 0.01 and ***p < 0.001 versus 0.

The cytotoxicity of the subfraction 3 (1.25–20 µg/mL) was tested on four different cell lines: human colorectal adenocarcinoma cell (HT‐29), human prostate carcinoma cell line (DU‐145), human neuroblastoma cell line (SH‐SY5Y) and human healthy fibroblast cell line (MRC‐5). The results from WST‐1 assay demonstrated that the subfraction 3 did not lead to cytotoxic effect on MRC‐5, unlike human cancer cells (HT‐29, DU‐145, SH‐SY5Y) (Figure 3). The results elucidated that the subfraction 3 from *T. annesophieae* is capable of killing cancerous cells even at low concentrations; however, its increasing concentrations exerted more anticancer effect, meaning that its anticancer action is dose‐dependent manner. For example, the maximum cytotoxic effect of the subfraction 3 on all cancer cell lines was detected at its dose of 20 µg/mL. These findings are consistent with those of the literature studies, showing that secondary metabolites from other *Talaromyces* species have the potential to be used as anticancer agents (Dong et al. 2006; Lan and Wu 2020; Hong et al. 2022; Tang et al. 2024). Anticancer effect was also confirmed by the abnormal changes in their cell morphologies induced by the sub‐fraction 3 (Supporting Information S1: Figure S5). The IC_50_ values of the subfraction 3 were determined to be 7.65, 3.76, and 10.76 µg/mL against HT‐29, DU‐145, and SH‐SY5Y, respectively (Figure 3). The IC_50_ values of the subfraction 3 are lower than the IC_50_ values of anticaner metabolites from other *Talaromyces* species in previous studies. For example, in a study (Dong et al. 2006), the metabolites from *T. wortmannii* were found to exhibit cytotoxic activity on human cancer cell lines (HCT‐5, HCT‐115, A549, MDA‐MB231, and K562) with IC_50_ values ranging from 28.7 to 130.5 μM. In another study (Hong et al. 2022), it was determined that a compound from *Talaromyces* sp. exhibited inhibitory effects on two gastric cancer cell lines (MGC803 and MKN28) with IC_50_ values of 71.8 and 122.7 μM, respectively.

**Figure 4:**
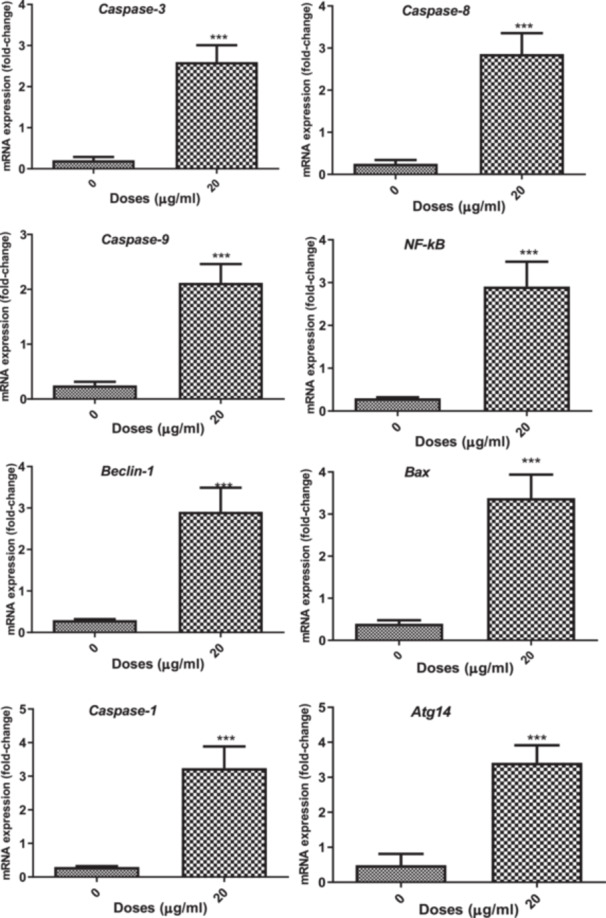
The changes in the gene expression levels of HT‐29 cells. Cells were treated with the fraction 3 (20 µg/mL) for 24 h. Control group was not treated with the fraction 3 (0 µg/mL). Two independent experiments were conducted and measurements were made in at least three replicates (*n* = 6) and presented as mean ± SD. ***p < 0.001 versus 0.

**Figure 5:**
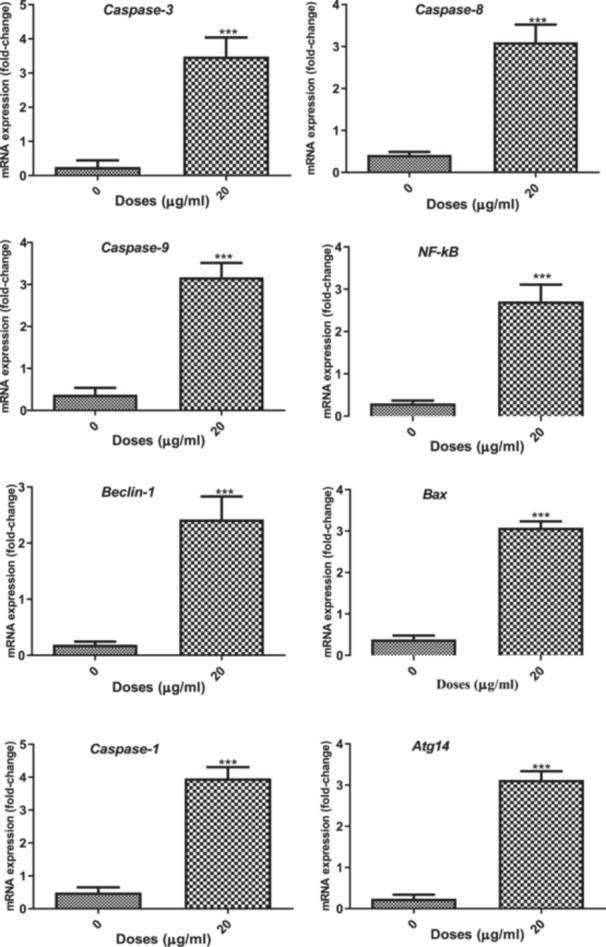
The changes in the gene expression levels of DU‐145 cells. Cells were treated with the fraction 3 (20 µg/mL) for 24 h. Control group was not treated with the fraction 3 (0 µg/mL). Two independent experiments were conducted and measurements were made in at least three replicates (*n* = 6) and presented as mean ± SD. ***p < 0.001 versus 0.

**Figure 6:**
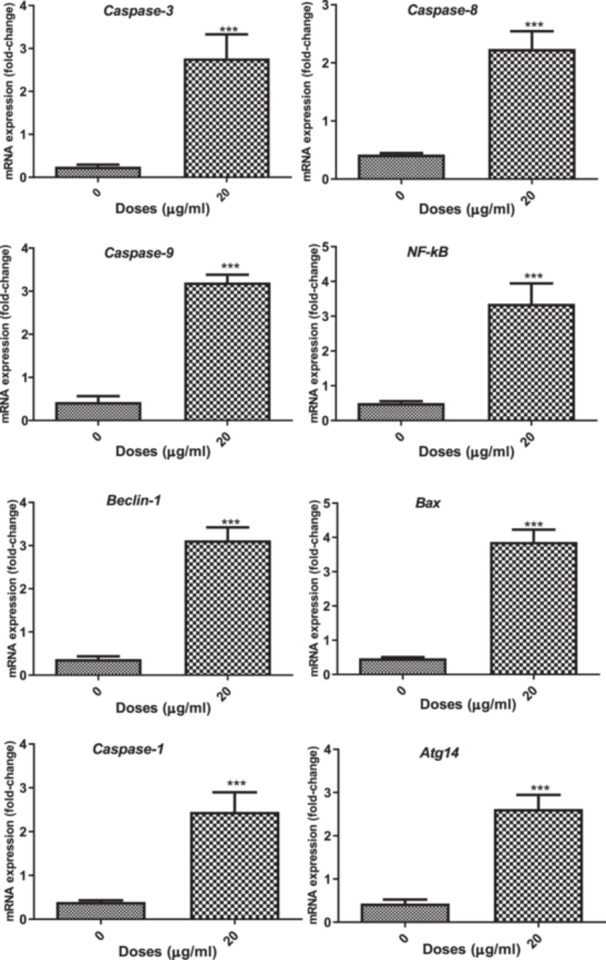
The changes in the gene expression levels of SH‐SY5Y cells. Cells in treatment group were treated with the fraction 3 (20 µg/mL) for 24 h. Control group was not treated with the fraction 3 (0 µg/mL). Two independent experiments were conducted and measurements were made in at least three replicates (*n* = 6) and presented as mean ± SD. ***p < 0.001 versus 0.

In this study, to elucidate the underlying mechanisms of the anticancer effect of the subfraction 3, expression analyses of some genes involved in apoptosis and autophagy were performed. The analyses were performed for control group and high dose fraction group (20 µg/mL). The results revealed that in comparison to the control, the subfraction 3 caused statistically significant increases in the expression levels of Casp3, Casp8, Casp9, NF‐kB, beclin‐1, bax, Casp1 and Atg14 genes in HT‐29 (Figure 4), DU‐145 (Figure 5) and SH‐SY5Y (Figure 6) cancer cell lines (*p* < 0.001). These results imply that treatment of cancer cells with the fraction 3 induced a pronounced upregulation of genes involved in apoptosis, autophagy, and inflammatory signaling relative to untreated controls. The concurrent increase in initiator caspases, caspase‐8 and caspase‐9, together with the executioner caspase‐3, indicates that both extrinsic, death receptor–dependent and intrinsic, mitochondrial apoptotic pathways are engaged (Sahoo et al. 2023; Dou et al. 2024). This effect is further reinforced by elevated Bax expression, a critical pro‐apoptotic regulator that promotes mitochondrial outer membrane permeabilization and facilitates cytochrome c–mediated activation of downstream caspases. At the same time, the observed induction of Beclin‐1 and Atg14 highlights activation of autophagic processes, as these proteins are central to autophagosome formation and early‐stage autophagy signaling. Considering the crosstalk between apoptosis and autophagy, the simultaneous upregulation of pro‐apoptotic and autophagy‐related genes suggests that the fraction 3 may tip the balance from cytoprotective autophagy toward a cell death–promoting response. Additionally, increased caspase‐1 expression underscores the involvement of inflammasome‐mediated signaling, which may contribute to inflammatory cell death or modulate stress responses in cancer cells. Although NF‐κB is traditionally associated with pro‐survival signaling, its elevated expression in this context likely represents a transient adaptive response to apoptotic and autophagic stress. Collectively, these findings demonstrate that the fraction 3 exerts multifaceted anticancer effects by coordinating apoptotic, autophagic, and inflammatory pathways, thereby undermining mechanisms that typically sustain cancer cell viability.

#### 3.4.3 Antioxidant Activity of Subfraction 3

The exogenous sources of natural compounds with antioxidant activity include fungi, plants, animals, bacteria and algae. *In vitro* assays are commonly used to assess the antioxidant capacity of natural compounds from these sources (Esim et al. 2024; Arslan et al. 2025a 2025c).

**Figure 7:**
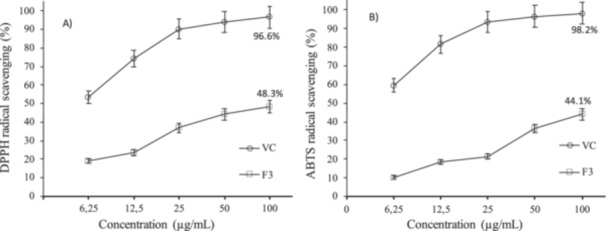
*In vitro* antioxidant potential of fraction 3 against DPPH (A) and ABTS (B) radicals. Two independent experiments were conducted and measurements were made in at least three replicates (*n* = 6) and presented as mean ± SD.

In the present study, DPPH and ABTS‐radical scavenging tests were used to evaluate the antioxidant and radical scavenging capacity of subfraction 3. The results displayed that the antioxidant capacity of the subfraction 3 increased proportionally in concentration‐depended manner and reached to the highest level at the concentration of 100 μg/mL. At this concentration, DPPH and ABTS radical scavenging activities of the subfraction 3 were determined as 48.3% and 44.1% (Figure 7). When compared to the positive control (Vitamin C), the antioxidant capacity of the subfraction 3 was moderate. These results suggest that the subfraction 3 may reduce oxidative stress via different mechanisms, supporting its possible protective role in biological systems.

#### 3.4.4 In Vitro Cytoprotective Effect of Subfraction 3

**Figure 8:**
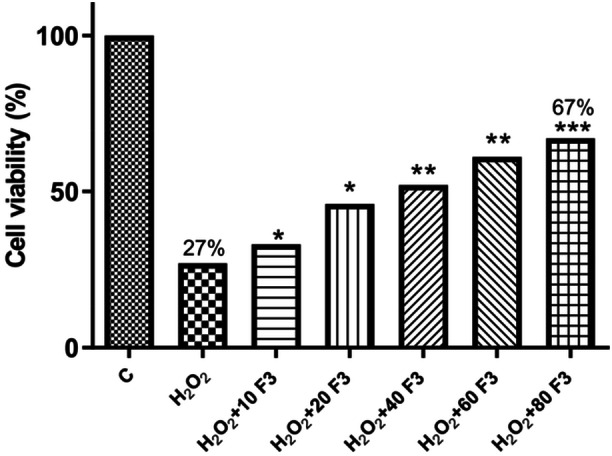
*In vitro* anti‐sking aging activity of fraction 3 in H_2_O_2_‐induced toxicity model. Data are given as mean ± standard deviation and *n* = 6. * *p* < 0.05 versus 0; ** *p* < 0.01 versus 0; *** *p* < 0.001 versus 0.

Since the fraction 3 exhibited *in vitro* antioxidant activities in DPPH and ABTS radical scavenging assays, we hypothesized that the subfraction 3 may also possess cytoprotective properties. To support this hypothesis, the study also focused on testing whether the fraction can protect normal human dermal fibroblasts (NHDFs) against H_2_O_2_ toxicity, as reported in earlier studies (Liu et al. 2022; Quiles et al. 2022; Tang et al. 2024). In the experimental procedure, NHDFs were first treated with the subfraction 3 (10–80 µg/mL) and then exposed to H_2_O_2_ toxicity. NHDFs that were not treated with the subfraction 3 or H_2_O_2_ were used as a control. The aim was to test the potential protective effect of the subfraction 3 against H_2_O_2_ toxicity. As shown in Figure 8, there was a significant decrease in density of NHDFs exposed to H_2_O_2_ compared to the control group (The final cell viability of NHDFs in H_2_O_2_‐treated group was foun to be 27%). On the contrary, the supplementation of the subfraction 3 (subfraction + H_2_O_2_ group) into the medium prior to H_2_O_2_ treatment was ascertained to reduce the toxic effect of H_2_O_2_ on NHDFs. In addition, it was determined that the protective effect of the subsfraction 3 increased as its concentration increased. Especially at the subfraction concentration of 80 µg/mL (subfraction 3 + H_2_O_2_ treatment group), the cell density of NHDFs was found to be significantly higher than H_2_O_2_ group (Figure 8). These findings suggest that the subfraction 3 or its bioactive components may be used as reliable and effective ingredients of dermocosmetics against chemical substance‐induced cytotoxicity.

### 3.5 Chemical Characterization of Subfraction 3

When the UV spectrum of the subfraction 3 was examined, the λmax value was observed to be in the 230–240 nm range. Since it is known that weak‐to‐moderate conjugated systems and aromatic compounds typically signal in the 230–240 nm region in UV light, while aliphatic compounds (λmax usually < 200 nm) mostly do not signal, it was assumed that the compounds in the fraction have aromatic properties (Supporting Information S1: Figure S6). Furthermore, when the HPLC chromatogram of the fraction 3 at 235 nm was examined, it was understood that this fraction contained at least four substances with close polarity. The HPLC findings at 235 nm also indicate the presence of nitrogen and oxygen‐containing conjugated systems, heteroaromatic rings, and UV‐active substances in the fraction. According to this chromatogram, it is understood that there are very few UV‐inactive compounds in the fraction, but there are many UV‐active, low molecular weight compounds with conjugated systems (Supporting Information S1: Figure S7).

Results obtained in LC‐MS positive ion mode revealed that the fraction contained compounds with high nitrogen content. For example, the compound C_5_H_3_O_4_N_8_ (m/z 239.02692) was identified as the most dominant ion with 100% relative intensity in positive mode (Supporting Information S1: Figure S9a–c and Table S3). The literature suggests that such molecular structures with high nitrogen content may be purine and pyrimidine derivatives (Alberts et al. 1994; Berg et al. 2002) and nitrogen‐rich heterocyclic metabolite (Dey et al. 2020; Kerru et al. 2020; De 2023; Shi et al. 2024). The complete absence of C_5_H_3_O_4_N_8_ in GC‐MS indicates that it has a polar and unstable heterocyclic structure. It is known that purine/pyrimidine derivatives exhibit their maximum UV absorbance around 250–280 nm (Kaspar et al. 2019). Whereas, this dominant compound resulted in maximum UV absorbance at 230–240 nm. This result minimizes the likelihood of C_5_H_3_O_4_N_8_ being a purine/pyrimidine derivative. For this compound, the dominant fragments observed in positive MS/MS analysis, such as m/z 179, 164, and 165, indicate that it has a conjugated and aromatic heterocyclic core (Supporting Information S1: Figure 9d‐f and Table S4). For example, this degradation pattern supports the idea that the compound C_5_H_3_O_4_N_8_ may be a nitrogen‐containing secondary metabolite (alkaloid or a nitrogen‐rich heterocyclic metabolite). It is known that alkaloids have a large carbon skeleton (C10‐C30) and contain 1–3 nitrogen atoms (purin alkaloids contain 4 nitrogen), while nitrogen‐rich heterocyclic metabolites have a small skeleton (C_4_–C_10_) and contain 4–10 nitrogen atoms (Dey et al. 2020; Kerru et al. 2020; De 2023). This information also indicates that C_5_H_3_O_4_N_8_ is a nitrogen‐rich heterocyclic metabolite rather than an alkaloid.

In negative ion mode, compounds with the molecular formulas C_9_H_9_O_4_N at m/z 195.05270, C_15_H_25_O_4_N_4_ at m/z 325.18683, and C_23_H_21_N_4_ at m/z 353.17584 were detected (Supporting Information S1: Figure S10a–f and Tables S5 and S6). However, the major compound was C_9_H_9_O_4_N. Since it was detected in the negative ion mode, it was concluded that this compound may have an acidic rather than a basic character, for example, it could be an aminobenzoic‐acid‐type or heteroaromatic carboxylic acid derivative.

Literature reports that aminobenzoic‐acid‐type or heteroaromatic carboxylic acid derivatives exhibit antimicrobial activity (Song et al. 2009; Laborda et al. 2018; Song et al. 2019; Rogala et al. 2019). Based on this information, it is possible to say that C_9_H_9_O_4_N is one of the compounds responsible for the antimicrobial activity of fraction 3. Considering the carbon and nitrogen numbers as described in the literature (Dey et al. 2020; Kerru et al. 2020; De 2023), C_23_H_21_N_4_ and C_15_H_25_O_4_N_4_ were concluded to be alkaloid‐like heterocyclic metabolites or nitrogen‐rich heterocyclic metabolites.

Overall, UV (230‐240 nm), HPLC (UV 235 nm), GC‐MS, LC‐MS, and MS/MS analyses reveal that the compounds in fraction 3 (C_5_H_3_O_4_N_8_, C_9_H_9_O_4_N, C_15_H_25_O_4_N_4_, and C_23_H_21_N_4_) are non‐volatile, polar, thermally unstable, and nitrogenous metabolites. Among them, three compounds (C_5_H_3_O_4_N_8_, C_15_H_25_O_4_N_4_, and C_23_H_21_N_4_) were concluded to be alkaloid‐like heterocyclic nitrogenous metabolites and/or nitrogen‐rich heterocyclic secondary metabolites (Table 2). However, further structural elucidation studies (NMR, HR‐MS/MS‐based structural verification) are needed for these compounds.

**Table 2:** Major compounds of fraction 3 based on chemical characterization.

LC‐MS/MS			GC‐MS	Description
Ion mode	m/z (MS)	Formula		
Positive (Dominant compound)	239.03	C_5_H_3_O_4_N_8_	Non detected	Non‐volatile, polar, thermally unstable, highly nitrogen‐rich heterocyclic structure
Negative (Dominant compound)	195.05	C_9_H_9_O_4_N	Non detected	Non‐volatile, polar, thermally unstable, oxygen‐rich, an aminobenzoic‐acid–type or heteroaromatic carboxylic acid derivative molecule with small‐size (< 300 Da)
Negative	325.19	C_15_H_25_O_4_N_4_	Non detected	Non‐volatile, polar, thermally unstable, a alkaloid‐like heterocyclic nitrogenous metabolite or nitrogen‐rich heterocyclic metabolite with medium‐sized (300‐700 Da)
Negative	353.18	C_23_H_21_N_4_	Non detected	Non‐volatile, polar, thermally unstable, alkaloid‐like heterocyclic nitrogenous metabolite or nitrogen‐rich heterocyclic metabolite with medium‐sized (300‐ 700 Da)

The literature reports that these metabolites can exhibit various biological activities such as antimicrobial, anti‐inflammatory, and anticancer properties (Zhang et al. 2018; Anh et al. 2022; Shi et al. 2024; Maji et al. 2025; Nehra et al. 2025). Their antimicrobial actions can be attributed to various mechanisms, such as interacting with microbial enzymes, nucleic acids, and membrane‐associated targets. Similarly, they exhibit anticancer effects by interacting with DNA (intercalation and groove binding), inhibiting enzymes (topoisomerases and protein kinases), causing excessive ROS production, regulating cellular signaling pathways, and triggering apoptosis (Seca and Pinto 2018; Kumar et al. 2023; Maji et al. 2025; Nehra et al. 2025; dos Santos et al. 2026). From a structure‐activity relationship (SAR) perspective, it is considered that high nitrogen content enables these molecules to bind stronger to proteins and DNA via H‐bonds and ionic interactions. In addition, the heteroaromatic ring mimics nucleobases, the planarity facilitates DNA intercalation, the substituent polarity affects solubility and selectivity, and the conjugated system stabilize π–π stacking with DNA bases (Kerru et al. 2020; Obernikhina et al. 2022; Evren et al. 2024; El‐Hema et al. 2026).

## 4 Conclusion

This study focused on the potential of locally isolated filamentous fungi *Talaromyces annesophieae* MD2 to produce bioactive secondary metabolites. The dichloromethane extract from the culture supernatant of the fungus was fractionated using chromatography methods. According to the bioactivity‐guided fractionation process, the subfraction 3 was selected as the principal fraction. The subfraction 3 exhibited broad‐spectrum antibacterial activity against both Gram‐positive and Gram‐negative bacteria and also caused selective cytotoxic actions on diverse cancer cell lines without affecting healthy cells. Furthermore, this subfraction displayed moderate antioxidant activity and conferred protection against H_2_O_2_‐induced cellular toxicity, supporting its cytoprotective potential. Structural and chemical analyses elucidated that the subfraction 3 contained four nitrogenous, moderately polar, non‐volatile, and thermally unstable metabolites, three of which are likely alkaloid‐like or nitrogen‐rich heterocyclic structures. Overall, the findings of this study suggest that the nitrogenous metabolites of filamentous fungus *T. annesophieae* MD2 may find potential applications in antimicrobial therapy, oncology, and dermocosmetic development. However, further studies are needed to individually purify and characterize each bioactive metabolite in the subfraction 3, and to fully elucidate its biological activities and underliying mechanisms.

## Author Contributions

**Meryem Doymus‐Yilmaz:** conceptualization, methodology, investigation, data curation, and writing – original draft. **Huseyin Aksit:** methodology and investigation. **Mesut Taskin:** methodology, data curation, supervision, writing – original draft and writing – review and editing.

## Ethics Statement

The authors have nothing to report.

## Conflicts of Interest

The authors declare no conflicts of interest.

## Supporting information


Supporting File

## Data Availability

The data that supports the findings of this study are available in the Supporting material of this article.
