# Supplementary material for: Water Soluble‐Nitrogenous Secondary Metabolites From Talaromyces annesophieae MD2 Exhibit Anti‐Bacterial, Anti‐Cancer, Anti‐Oxidant, and Cytoprotective Activities
Source: Microbiologyopen. 2026 Jul 19;15(4):e70367. doi: 10.1002/mbo3.70367 (PMC13382376; doi:10.1002/mbo3.70367)
Supplement: Supplementary file 1 — Supporting File [file MBO3-15-e70367-s001.docx]

**Water soluble-nitrogenous secondary metabolites from *Talaromyces annesophieae* MD2 exhibit anti-bacterial, anti-cancer, anti-oxidant, and cytoprotective activities**

**Supplementary Material**

# **Supplementary Table 1.** Genes and Primers Used in the Study

| **Gene** | **Forward Primer (5’→3’)** | **Reverse Primer (5’→3’)** |
| --- | --- | --- |
| β-actin | AGAGCTACGAGCTGCCTGAC | AGCACTGTGTTGGCGTACAG |
| Caspase-3 | TGTCATCTCGCTCTGGTACG | AAATGACCCCTTCATCACCA |
| Caspase-8 | ATGAAAAGCAAACCTCGGGG | TTCAGGTACTTTCAGGAGGC |
| Caspase-9 | CAGATGCTGTCCCATAGACG | CAGGACACAAGGCTGATGGA |
| Caspase-1 | GGGTTGGTGATGATGTGGCT | CCAGGACAGTGGAGTTGTTG |
| NF-κB | AAGTGATCCCTTATTGCCCAG | TTGGTGGGTGCGTCTTAGTT |
| Beclin-1 | GATGACAGTGGGGAGGAAGAG | GCCTGGGCTGTGGTAAGTA |
| Bax | TTGCTTCAGGGTTTCATCCA | GATCAGCTCGGGCACTTTAG |
| Atg14 | GAGATCCAGCTGAACTGGGA | TTGGCTGGTGTGTTTTCTCC |


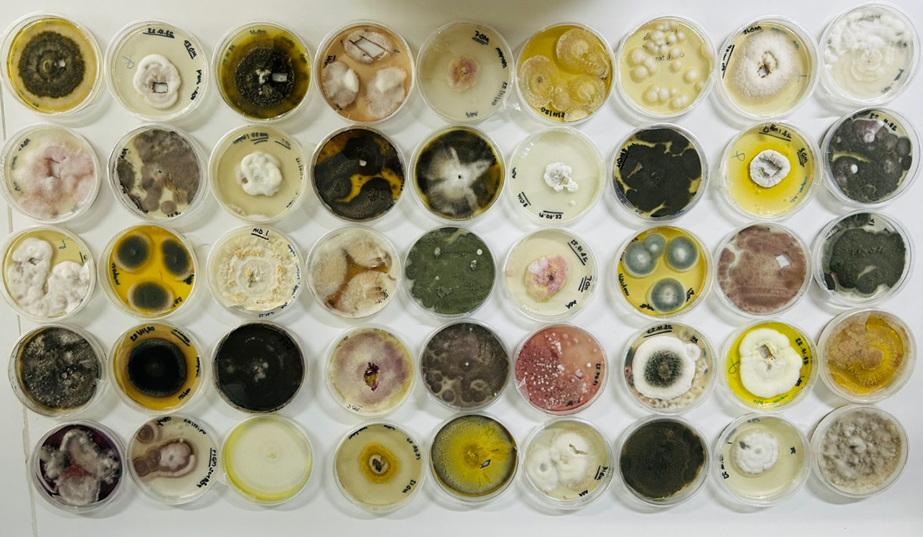


**Supplementary Fig. 1.** Mycelial morphological of 40 fungal strains from different soil samples

**Supplementary Table 2.** **Antimicrobial activity of fungal extracts**

| Isolation source | Isolate | Zone diameter (mm) | | Isolation source | Isolate | Zone diameter (mm) | |
| --- | --- | --- | --- | --- | --- | --- | --- |
|  |  | *S. aureus* | *E. coli* |  |  | *S. aureus* | *E. coli* |
| Pine forest soil (Palandöken, Erzurum) | **MD 1** | - | - | **Pine forest soil (Pasinler, Erzurum)** | **MD 21** | - | - |
|  | **MD 2** | 17±1.0 | 22±1.0 |  | **MD 22** | - | - |
|  | **MD 3** | - | - |  | **MD 23** | - | - |
|  | **MD 4** | - | - |  | **MD 24** | 16±1.0 | - |
|  | **MD 5** | - | - |  | **MD 25** | - | - |
|  | **MD 6** | - | - |  | **MD 26** | - | - |
|  | **MD 7** | - | 16±1.0 |  | **MD 27** | - | - |
|  | **MD 8** | 14±1.0 | - |  | **MD 28** | - | - |
|  | **MD 9** | - | - |  | **MD 29** | - | - |
|  | **MD 10** | - | - |  | **MD 30** | - | - |
| Pine forest soil  (Yakutiye, Erzurum) | **MD 11** | - | 15±1.0 |  | **MD 31** | - | - |
|  | **MD 12** | 15±1.0 | - |  | **MD 32** | - | - |
|  | **MD 13** | - | - |  | **MD 33** | - | - |
|  | **MD 14** | - | - | **Pine forest soil (Tortum, Erzurum)** | **MD 34** | - | - |
|  | **MD 15** | - | - |  | **MD 35** | - | - |
|  | **MD 16** | - | - |  | **MD 36** | - | - |
|  | **MD 17** | - | - |  | **MD 37** | - | - |
|  | **MD 18** | - | - |  | **MD 38** | - | - |
|  | **MD 19** | - | - |  | **MD 39** | - | - |
|  | **MD 20** | - | - |  | **MD 40** | - | - |

**Supplementary Fig.2.** Antimicrobial activity of some fungal isolates against *S. aureus* and *E. coli*


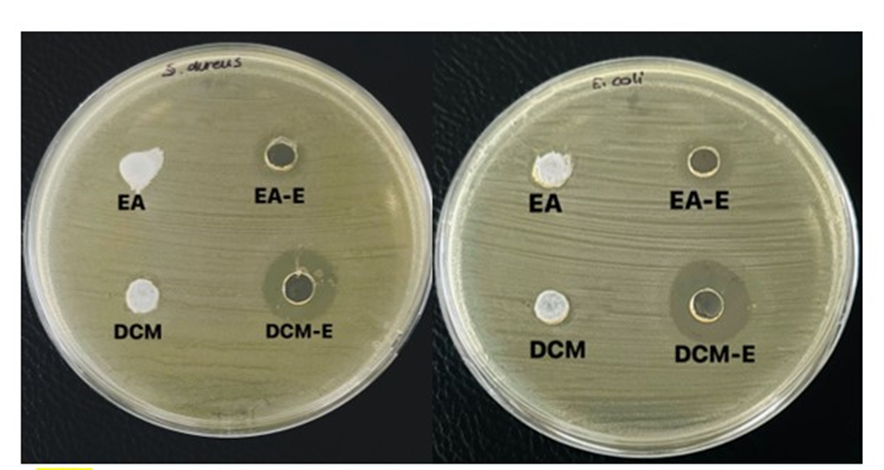


**Supplementary Fig 3.** Antimicrobial activity of different extracts from culture superntants of the isolate MD2 *(Talaromyces annesophieae).* *DCM-E* dichloromethane extract and *EA-E* ethyl acetate extract.

**Supplementary Fig. 4.** Antimicrobial activity of fraction 3 according to agar well diffusion assay method.


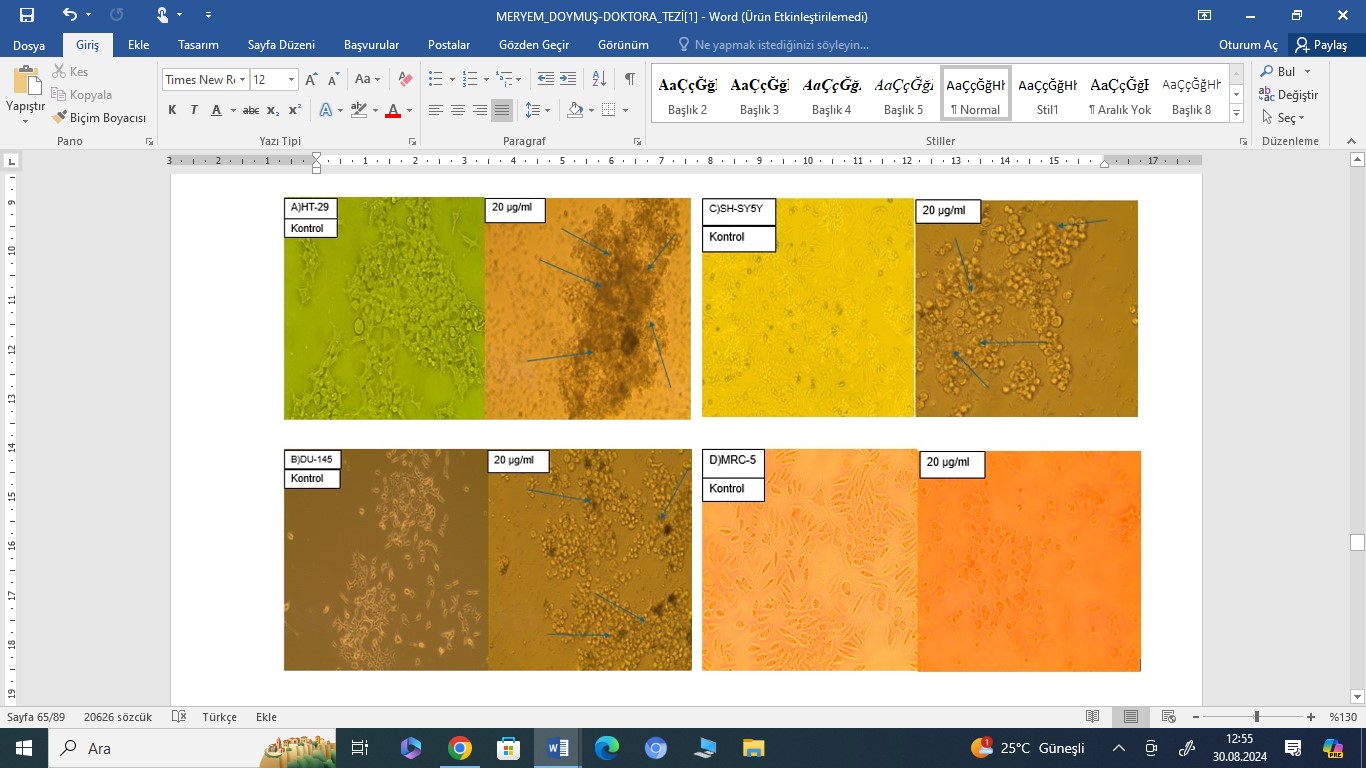


###### **Suplementary Fig. 5.** Morphological effects of Fraction 3 on HT-29 (A), DU-145 (B), SH-SY5Y (C) and (D) MRC-5 cell lines. No treatment was given to the control group.

Supplementary Fig. 6. UV spectrum of the fraction 3 (in methanol)

Supplementary Fig. 7. HPLC-UV chromatogram of the fraction (235 nm)


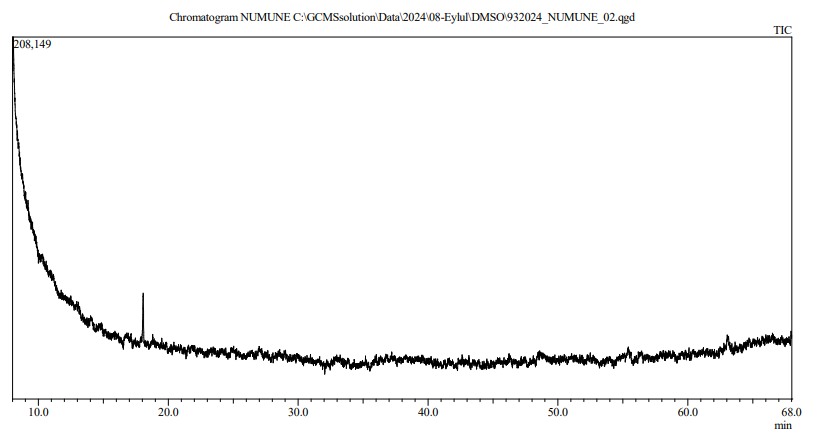


Supplementary Fig. 8**.** GC-MS chromatogram of the fraction 3

Supplementary Fig. 9a-c**.**

pozitif MS


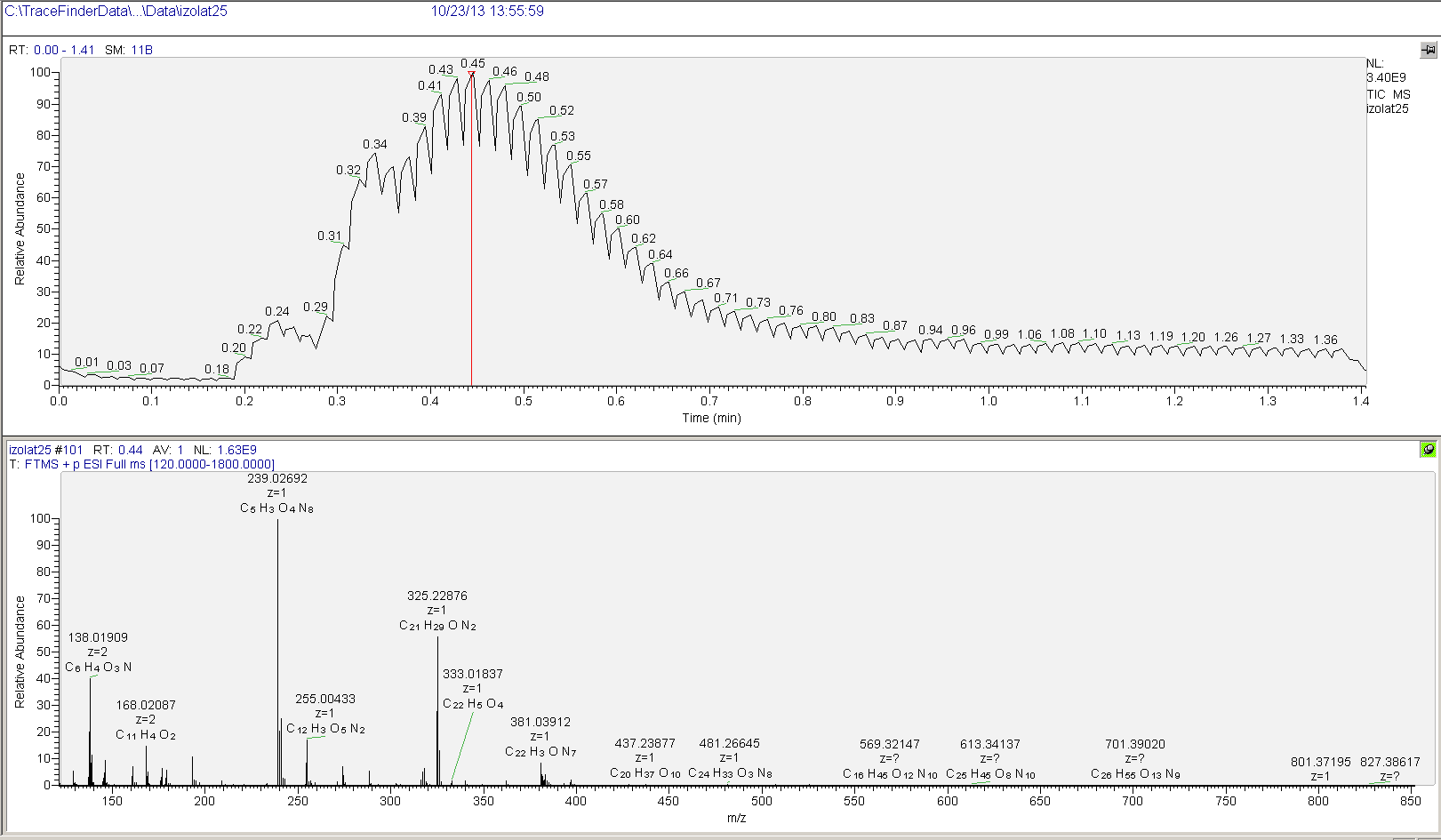

**Supplementary Table 3.**


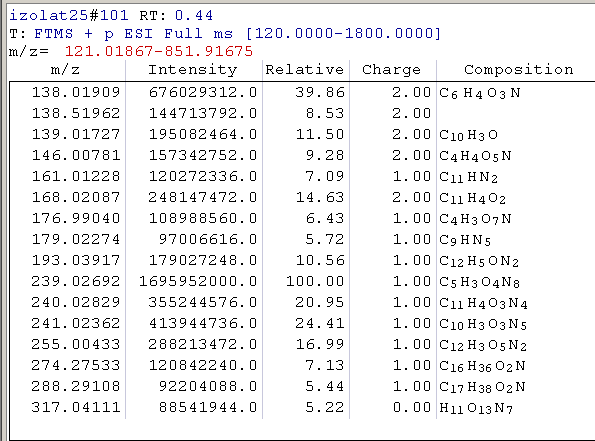


m/z Intensity Relative Charge Composition

138.01909 676029312.0 39.86 2.00 C6 H4 O3 N

138.51962 144713792.0 8.53 2.00

139.01727 195082464.0 11.50 2.00 C10 H3 O

146.00781 157342752.0 9.28 2.00 C4 H4 O5 N

161.01228 120272336.0 7.09 1.00 C11 H N2

168.02087 248147472.0 14.63 2.00 C11 H4 O2

176.99040 108988560.0 6.43 1.00 C4 H3 O7 N

179.02274 97006616.0 5.72 1.00 C9 H N5

193.03917 179027248.0 10.56 1.00 C12 H5 O N2

239.02692 1695952000.0 100.00 1.00 C5 H3 O4 N8

240.02829 355244576.0 20.95 1.00 C11 H4 O3 N4

241.02362 413944736.0 24.41 1.00 C10 H3 O3 N5

255.00433 288213472.0 16.99 1.00 C12 H3 O5 N2

274.27533 120842240.0 7.13 1.00 C16 H36 O2 N

288.29108 92204088.0 5.44 1.00 C17 H38 O2 N

317.04111 88541944.0 5.22 0.00 H11 O13 N7

318.30164 109497904.0 6.46 0.00 C21 H38 N2

325.22876 913204864.0 53.85 1.00 C21 H29 O N2

326.23193 212742320.0 12.54 1.00 C18 H32 O4 N

381.03912 149132896.0 8.79 1.00 C22 H3 O N7

pozitif MS/MS

Supplementary Fig. 9d-f**.**


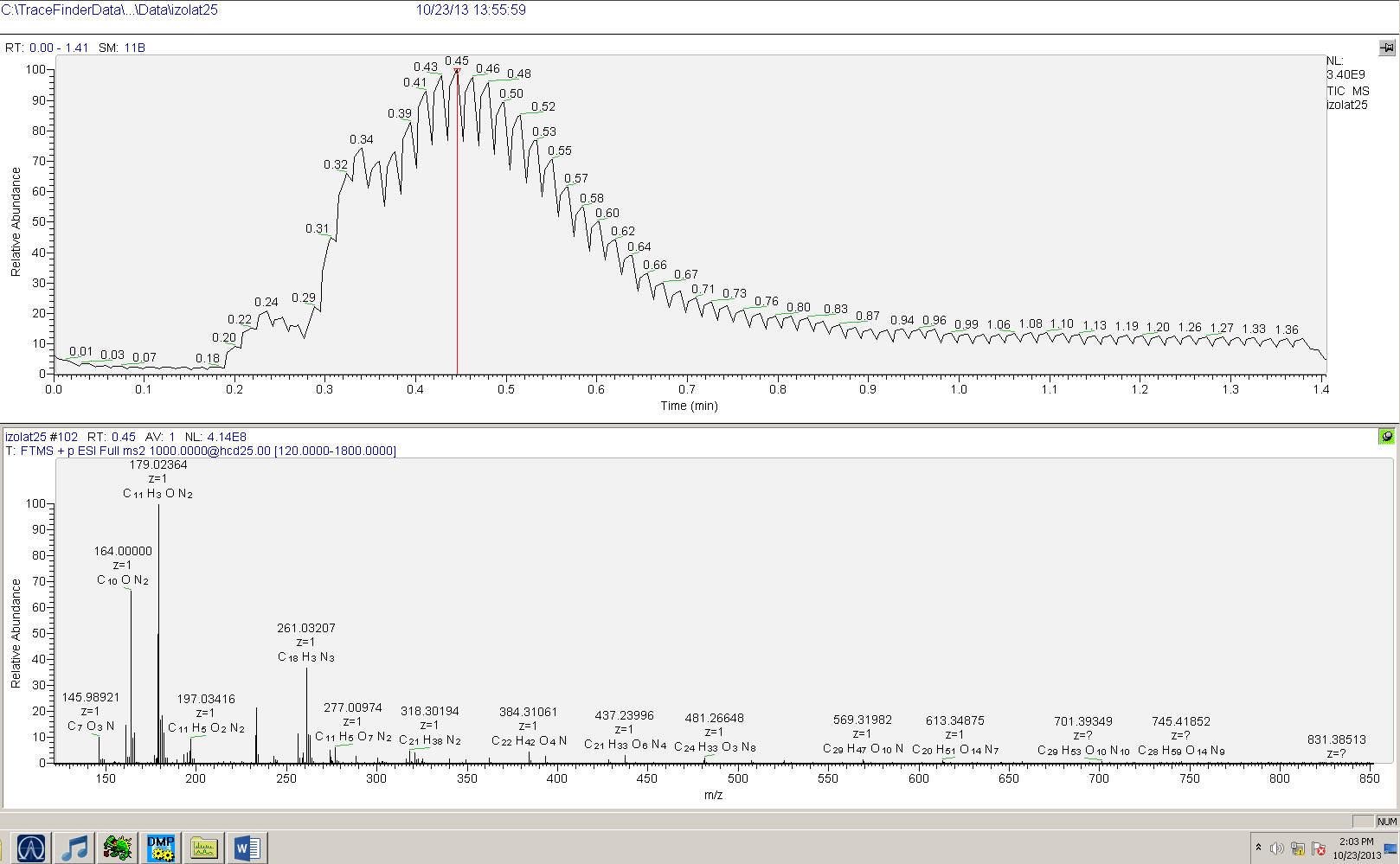

**Supplementary Table 4**


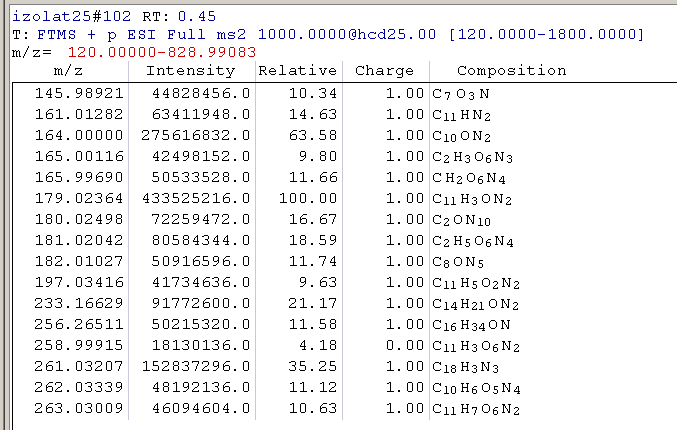


m/z Intensity Relative Charge Composition

145.98921 44828456.0 10.34 1.00 C7 O3 N

161.01282 63411948.0 14.63 1.00 C11 H N2

164.00000 275616832.0 63.58 1.00 C10 O N2

165.00116 42498152.0 9.80 1.00 C2 H3 O6 N3

165.99690 50533528.0 11.66 1.00 C H2 O6 N4

179.02364 433525216.0 100.00 1.00 C11 H3 O N2

180.02498 72259472.0 16.67 1.00 C2 O N10

181.02042 80584344.0 18.59 1.00 C2 H5 O6 N4

182.01027 50916596.0 11.74 1.00 C8 O N5

197.03416 41734636.0 9.63 1.00 C11 H5 O2 N2

233.16629 91772600.0 21.17 1.00 C14 H21 O N2

256.26511 50215320.0 11.58 1.00 C16 H34 O N

258.99915 18130136.0 4.18 0.00 C11 H3 O6 N2

261.03207 152837296.0 35.25 1.00 C18 H3 N3

262.03339 48192136.0 11.12 1.00 C10 H6 O5 N4

263.03009 46094604.0 10.63 1.00 C11 H7 O6 N2

274.27573 23409156.0 5.40 1.00 C16 H36 O2 N

277.00974 27853736.0 6.42 1.00 C11 H5 O7 N2

318.30194 19958772.0 4.60 1.00 C21 H38 N2

384.31061 19371436.0 4.47 1.00 C22 H42 O4 N

Supplementary Fig. 10a-c**.**

NEGATIVE MS


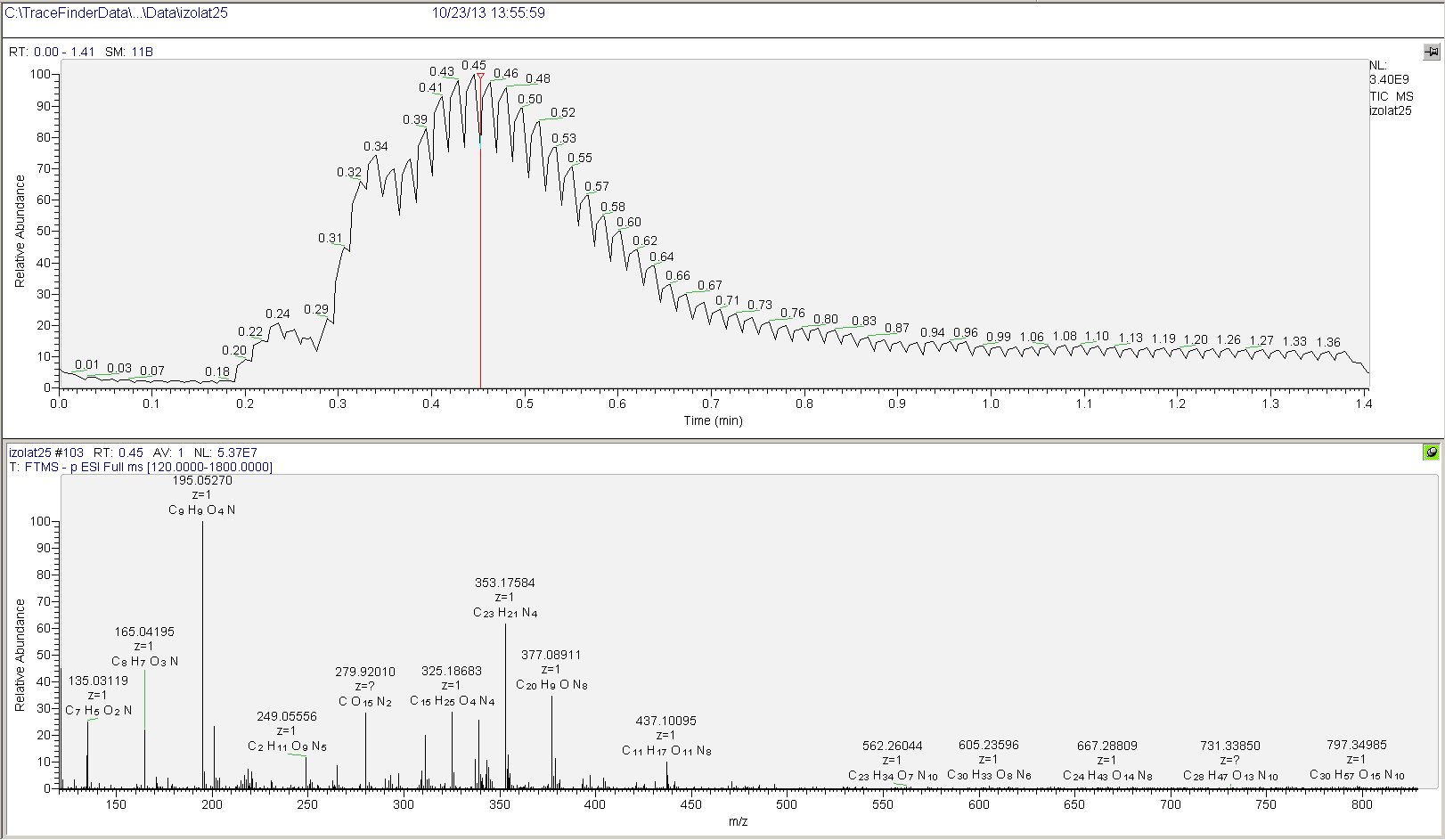

**Supplementary Table 5**


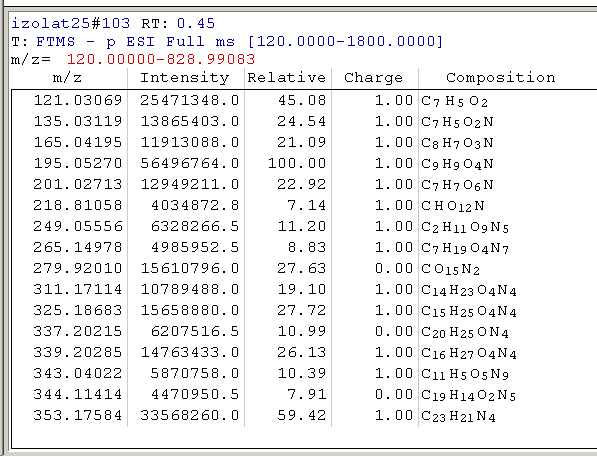


m/z Intensity Relative Charge Composition

121.03069 25471348.0 45.08 1.00 C7 H5 O2

135.03119 13865403.0 24.54 1.00 C7 H5 O2 N

165.04195 11913088.0 21.09 1.00 C8 H7 O3 N

195.05270 56496764.0 100.00 1.00 C9 H9 O4 N

201.02713 12949211.0 22.92 1.00 C7 H7 O6 N

218.81058 4034872.8 7.14 1.00 C H O12 N

249.05556 6328266.5 11.20 1.00 C2 H11 O9 N5

265.14978 4985952.5 8.83 1.00 C7 H19 O4 N7

279.92010 15610796.0 27.63 0.00 C O15 N2

311.17114 10789488.0 19.10 1.00 C14 H23 O4 N4

325.18683 15658880.0 27.72 1.00 C15 H25 O4 N4

337.20215 6207516.5 10.99 0.00 C20 H25 O N4

339.20285 14763433.0 26.13 1.00 C16 H27 O4 N4

343.04022 5870758.0 10.39 1.00 C11 H5 O5 N9

344.11414 4470950.5 7.91 0.00 C19 H14 O2 N5

353.17584 33568260.0 59.42 1.00 C23 H21 N4

354.17923 6956128.5 12.31 1.00 C18 H22 O2 N6

377.08911 18689580.0 33.08 1.00 C20 H9 O N8

379.08633 6419902.0 11.36 1.00 C27 H11 O N2

437.10095 5598959.5 9.91 1.00 C11 H17 O11 N8

NEGATIF MS/MS

Supplementary Fig. 10d-f


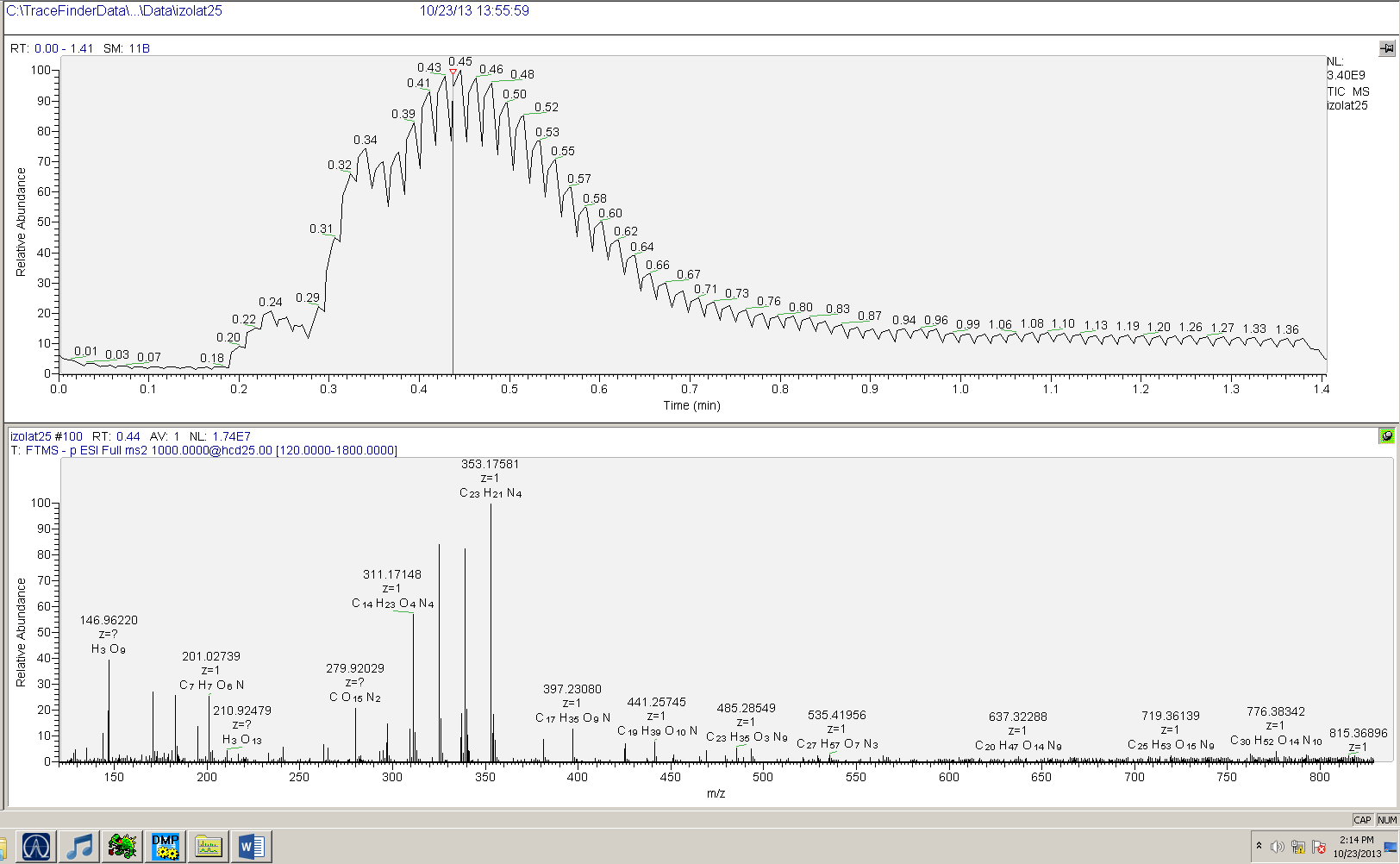

**Supplementary Table 6**


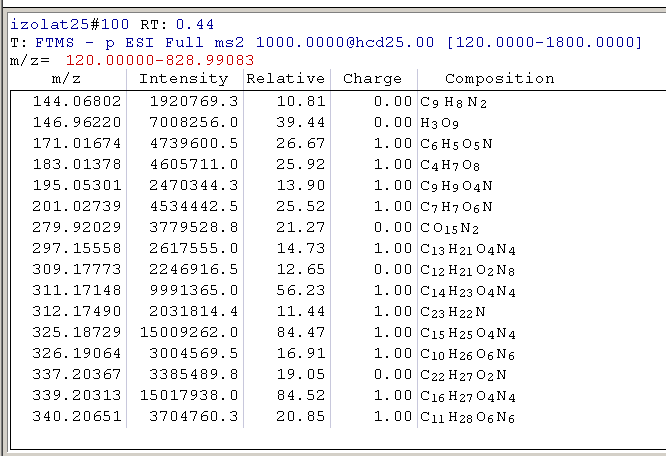


m/z Intensity Relative Charge Composition

144.06802 1920769.3 10.81 0.00 C9 H8 N2

146.96220 7008256.0 39.44 0.00 H3 O9

171.01674 4739600.5 26.67 1.00 C6 H5 O5 N

183.01378 4605711.0 25.92 1.00 C4 H7 O8

195.05301 2470344.3 13.90 1.00 C9 H9 O4 N

201.02739 4534442.5 25.52 1.00 C7 H7 O6 N

279.92029 3779528.8 21.27 0.00 C O15 N2

297.15558 2617555.0 14.73 1.00 C13 H21 O4 N4

309.17773 2246916.5 12.65 0.00 C12 H21 O2 N8

311.17148 9991365.0 56.23 1.00 C14 H23 O4 N4

312.17490 2031814.4 11.44 1.00 C23 H22 N

325.18729 15009262.0 84.47 1.00 C15 H25 O4 N4

326.19064 3004569.5 16.91 1.00 C10 H26 O6 N6

337.20367 3385489.8 19.05 0.00 C22 H27 O2 N

339.20313 15017938.0 84.52 1.00 C16 H27 O4 N4

340.20651 3704760.3 20.85 1.00 C11 H28 O6 N6

353.17581 17768126.0 100.00 1.00 C23 H21 N4

354.17899 3322072.0 18.70 1.00 C17 H26 O6 N2

381.23514 1562248.6 8.79 1.00 C16 H29 O3 N8

397.23080 2280123.3 12.83 1.00 C17 H35 O9 N
